# Improving crop salt tolerance through soil legacy effects

**DOI:** 10.3389/fpls.2024.1396754

**Published:** 2024-05-10

**Authors:** Yue Ma, Chunyan Zheng, Yukun Bo, Chunxu Song, Feng Zhu

**Affiliations:** ^1^ Key Laboratory of Agricultural Water Resources, Hebei Key Laboratory of Soil Ecology, Center for Agricultural Resources Research, Institute of Genetics and Developmental Biology, Chinese Academy of Sciences, Shijiazhuang, China; ^2^ University of Chinese Academy of Sciences, Beijing, China; ^3^ State Key Laboratory of Nutrient Use and Management, College of Resources and Environmental Sciences, National Academy of Agriculture Green Development, China Agricultural University, Beijing, China; ^4^ Key Laboratory of Plant-Soil Interactions, Ministry of Education, China Agricultural University, Beijing, China; ^5^ National Observation and Research Station of Agriculture Green Development, Quzhou, China

**Keywords:** saline soil, halophytes, salt-tolerant crops, beneficial microorganism, salt tolerance

## Abstract

Soil salinization poses a critical problem, adversely affecting plant development and sustainable agriculture. Plants can produce soil legacy effects through interactions with the soil environments. Salt tolerance of plants in saline soils is not only determined by their own stress tolerance but is also closely related to soil legacy effects. Creating positive soil legacy effects for crops, thereby alleviating crop salt stress, presents a new perspective for improving soil conditions and increasing productivity in saline farmlands. Firstly, the formation and role of soil legacy effects in natural ecosystems are summarized. Then, the processes by which plants and soil microbial assistance respond to salt stress are outlined, as well as the potential soil legacy effects they may produce. Using this as a foundation, proposed the application of salt tolerance mechanisms related to soil legacy effects in natural ecosystems to saline farmlands production. One aspect involves leveraging the soil legacy effects created by plants to cope with salt stress, including the direct use of halophytes and salt-tolerant crops and the design of cropping patterns with the specific crop functional groups. Another aspect focuses on the utilization of soil legacy effects created synergistically by soil microorganisms. This includes the inoculation of specific strains, functional microbiota, entire soil which legacy with beneficial microorganisms and tolerant substances, as well as the application of novel technologies such as direct use of rhizosphere secretions or microbial transmission mechanisms. These approaches capitalize on the characteristics of beneficial microorganisms to help crops against salinity. Consequently, we concluded that by the screening suitable salt-tolerant crops, the development rational cropping patterns, and the inoculation of safe functional soils, positive soil legacy effects could be created to enhance crop salt tolerance. It could also improve the practical significance of soil legacy effects in the application of saline farmlands.

## 1 Introduction

In recent years, land degradation caused by climate change has posed a huge challenge to agricultural production. In the absence of major technological breakthroughs in agriculture, existing arable land resources are hardly sufficient to support global food security (German et al., 2017; Hartmann and Six, 2023). Saline farmland is an important reserve resource of arable land with great potential for ensuring food security and sustainable agricultural development (Negacz et al., 2022). Therefore, finding solutions to increase the productivity of saline farmland and improve crop tolerance to saline stress has become an important research topic currently (Munns and Tester, 2008).

Soil salinization is a global environmental problem, with more than 833 million hectares of soil and more than 10% of farmland affected by salinization (FAO, 2021), causing at least 25% of crops to suffer from varying degrees of yield loss due to persistent salt stress, with a serious impact on food security (Farooq et al., 2017; Kumar et al., 2022). Soil salinization leads to reduced crop yield because the significant negative impacts on seed germination by disrupting the membrane permeability of the seed embryo and increasing the osmotic stress on seeds (Deng et al., 2014). For salt-sensitive crops, seed germination rate, germination time, and the length of the plumule are all affected by salt stress (Abbas et al., 2012). Persistent salt stress during the crop growth phase leads to crop water loss and ion toxicity due to increased cellular osmotic pressure and disruption of cell membranes (Läuchli and Grattan, 2007). Salt stress also reduces nutrient uptake by inhibiting crop root growth (Burssens et al., 2000; West et al., 2004), inhibits photosynthesis by decreasing the crop’s leaf area (Hu et al., 2022), and ultimately affects crop yield and quality.

Moreover, the survival of microorganisms is directly associated with plant and soil environments (Pulleman et al., 2012). Salt stress can reduce the abundance and activity of soil microbial communities (Rietz and Haynes, 2003), affecting the composition of functional soil microbes (Zhang et al., 2019), and disrupting the stability of microbial networks (Li et al., 2023a). This disruption affects nutrient cycling (Bai et al., 2012) and material utilization (Elmajdoub and Marschner, 2013) ultimately affecting the ecological functions of soil microbial communities (Zhang et al., 2023). Weakened ecological functions of microbial community, in turn, affect plant-microbe interactions (Etesami and Beattie, 2017), as manifested by reduced microbial colonization (Li et al., 2023a) and impaired plant growth (Jansson et al., 2023).

Both plants and soil microorganisms have developed specific abilities and mutualistic associations to cope with various stresses (Zhao et al., 2020; Liu et al., 2022). Halophytes and salt-tolerant plants, as the dominant vegetation in saline environments, are better adapted to saline stresses and have formed unique strategies improving their adaptability through such pathways as salt gland excretion (Yuan F. et al., 2016), ionic and osmotic regulation (Zhu, 2016), antioxidant defenses (Apse and Blumwald, 2002) and root structural modifications (Yu et al., 2022). Soil microorganisms also have various salt-tolerance strategies, such as salt accumulation and synthesis of organic osmotic material to adapt to high-salt environments (Gunde-Cimerman et al., 2018). Meanwhile, beneficial microorganisms can influence performance of their host plants under harsh conditions (Wang and Song, 2022). For example, arbuscular mycorrhizal fungi can help host plants to cope with abiotic stresses like drought, salt, etc., by improving plant water utilization, regulating photosynthesis and maintaining osmotic balance (Borde et al., 2017).

In addition, soil legacy effects are microbiological and functional substance traits retained in the soil by the plants, which influence the growth of succeeding plants (Van der Putten et al., 2013). The formation of soil legacy effects is the process of plant-microbe interactions in which plants respond to stressful stimuli and mobilize the required metabolites and functional microorganisms, thus promoting the growth of their own and succeeding plants as well as increasing their tolerance (Bakker et al., 2018). So, the application of soil legacy effects may also help to refine the way we cultivate and manage crops for agricultural production (Mariotte et al., 2018; Carrión et al., 2019; Cordovez et al., 2019). Therefore, based on the theoretical foundation of soil legacy effects in natural ecosystems, it is important to further explore the mechanism of crop-soil-microbe interactions in saline farmlands, which has profound implication for mitigating crop salt stress, increasing crop productivity and improving the environment of saline farmlands (Vukicevich et al., 2016).

## 2 Formation and role of soil legacy effects in natural ecosystems

In natural ecosystems, plants and soil organisms have various effects to soil legacy (Wardle et al., 2004; Faucon et al., 2017). Plant species with different root structures, growth habits and ways of interacting with soil organisms have important impacts on soil legacy effects (Oliver et al., 2021), while plant species composition and diversity also significantly modify such effects at the community level (Kowalchuk et al., 2002; Lange et al., 2015). Soil organisms, playing important roles in soil ecosystems, influence soil legacy effects by affecting soil organic matter decomposition, nutrient cycling and soil structure (Bardgett and Wardle, 2010).

Therefore, natural ecosystems have become a ‘database’ for exploring the mechanisms of soil legacy effects in the context of a highly diversified plants, microorganisms and soil environmental factors. An increasing number of studies have been carried out on the growth characteristics, resource utilization and survival strategies of plants and microorganisms that contribute to a better understanding about the soil legacy effects (Bezemer et al., 2006; Cortois et al., 2016; Bezemer et al., 2018; Heinen et al., 2020). The diversity of plant species, plant functional traits and soil microorganisms in natural ecosystems contributes to extensive research on species interactions and stress adaptations. The intricate interactions between plants and soil microorganisms play a crucial role in promoting the stabilization of soil ecosystems (Grayston et al., 1998; Berg and Smalla, 2009; Berendsen et al., 2012). Above- and below-ground interactions of plants have long-term legacy effects on biotic stresses in natural ecosystems and can improve plant performance and resistance by manipulating soil microbial communities (Wurst and Ohgushi, 2015; Pineda et al., 2017). For abiotic stresses, soil microorganisms are able to implement a variety of mechanisms to fight against them and keep soil fertility as well as plant development in good condition (Abdul Rahman et al., 2021). For example, drought stress-induced dominance of fungal communities can influence succeeding plant drought adaptation by maintaining higher rates of litter decomposition and soil respiration (Mariotte et al., 2015). Inoculation of drought-conditioned phyllosphere and soil microbial communities can make plants capable of coping with repeated drought stress (Li et al., 2022).

Plant functional group is a common concept in the study of soil legacy effects, which refers to a group of plants that respond similarly to ecological processes and environmental changes, such as the grasses, forbs and legumes that are frequently mentioned in the literature (Kulmatiski et al., 2008; Cortois et al., 2016). Different plant functional groups can create positive or negative soil legacy effects by accumulating soil pathogens, recruiting beneficial microorganisms and regulating interactions with insects, etc (Petermann et al., 2008; Latz et al., 2012; Heinen et al., 2019). Such soil legacy effects, mediated by aboveground plant functional groups and soil microorganisms, play a role for succeeding plant growth in terms of soil physical properties, soil nutrient availability, soil microbial community structure, stress tolerance and competitive coexistence relationships (Byun et al., 2013; Strecker et al., 2015; Fischer et al., 2018; Mackie et al., 2018; Adomako and Yu, 2023).

Different plant functional groups play distinct roles in shaping soil legacy effects. For instance, grasses may improve soil physical structure and water retention through dense root systems (Hanamant et al., 2022), while legumes retain soil nutrients through nitrogen fixation (Spehn et al., 2002). The soil legacy effects resulting from these changes in the soil environment create more favorable conditions for succeeding plant growth. Simultaneously, the interaction between various plant functional groups and soil microorganisms yields diverse soil legacy effects. Grasses and forbs secrete different carbon compounds into the soil, recruiting different soil microorganisms (Philippot et al., 2013). For example, the presence of the grasses *Lolium perenne* not only increased the density of active bacteria in the soil but also elevated the expression of biocontrol genes associated with these bacteria, thereby contributing to the productivity of succeeding plant communities (Latz et al., 2015). Moreover, grasses positively influence other plant functional groups by altering soil microbial communities and soil nutrients (Cortois et al., 2016). Forbs, however, with more decomposers and higher concentrations of chemicals in their litter, may negatively impact succeeding plants (Bonanomi et al., 2006).

To foster positive soil legacy effects, it is essential to manage specific plant functional groups, regulate appropriate levels of beneficial microorganisms, decomposers and pathogenic microorganisms, and develop diverse plant-microbe community interactions (Carrión et al., 2019; De la Fuente Cantó et al., 2020; Xiong et al., 2020; Song et al., 2021). However, there is a current lack of studies exploring the application of the principle of soil legacy effects in understanding plant salt tolerance. Most studies have focused on the mechanism of plant’s intrinsic salt tolerance and the utilization of specific microorganisms to enhance salt tolerance in laboratory and simulation experiments (Li et al., 2020a; Li et al., 2020b; Li et al., 2021a; Schmitz et al., 2022). Therefore, it is important to address how the rules of soil legacy effects can be developed and applied in saline farmlands.

## 3 Processes of plant response to salt stress

Plants have various strategies to cope with salt stress, involving refinement in their cellular physiology, phenotypic structures, osmoregulation, antioxidant production, and the regulation of signaling pathways (Van Zelm et al., 2020; Zhao et al., 2020). For instance, plants eliminate excess salt through a salt excretion mechanism to minimize salt-damage (Dassanayake and Larkin, 2017). Plants can also modify their root structure, such as developing deeper root systems to increase water uptake and mitigate the impact of salinity (Galvan-Ampudia and Testerink, 2011). In addition, plants respond to salt stress-induced damage by producing antioxidants, osmotic substances and protective enzymes (Hasegawa et al., 2000). ABA-dependent protein kinases are activated in response to salt stress, affecting cellulose distribution, controlling root tip cells, thus promoting salt avoidance in plant (Yu et al., 2022). Plant roots also secrete peptides that are transferred to the leaves to induce ABA accumulation, thereby driving stomatal closure to prevent leaf (Takahashi et al., 2018; Yu et al., 2020). Therefore, the combined application of these strategies enables plants to better adapt and survive in high-salt environments.

Besides plant innate responses, the complex microbial communities in rhizosphere soil play a critical role in host performance and tolerance to stresses (Durán et al., 2018; Carrión et al., 2019). These microbial communities help plants adapt to harsh conditions by forming mutualistic relationships, participating in nutrient uptake, producing beneficial compounds, and inducing immune responses that support plants against stress (Hou et al., 2021).

In terms of salinity tolerance, microorganisms establish mutually beneficial symbiotic relationships with plants through various mechanisms, assisting them in adapting to high salt environments. Rhizosphere microorganisms can secrete specific compounds, such as bacterial exopolysaccharides (EPS), which improve plant ion balance, promote soil aggregation, and thus maintain plant growth in high-salt (Morcillo and Manzanera, 2021). Arbuscular mycorrhizal fungi (AMF) enhance host plant salt tolerance by manipulating the osmotic balance through mycelium, improving access to water and nutrients (Hammer et al., 2011; Ruiz-Lozano et al., 2012). Moreover, rhizosphere microorganisms also play a role in physiological regulation and defense processes (Mishra et al., 2021). Plant growth promoting rhizobacteria (PGPR) can stimulate root development and enhance nutrient utilization under salt stress. For instance, the IAA-overproducing strain *Sinorhizobium meliloti* has been found to enhancive salt tolerance of alfalfa in saline soils by stimulating root proliferation (Bianco and Defez, 2009). Under salt stress conditions, the increase in the number and weight of root nodules in *Acacia gerrardii* inoculated with *Bacillus subtilis* contributed to the enhancement of nitrogen fixation by the roots, as well as uptake and systemic translocation of phosphorus by the plant (Hashem et al., 2016a, Hashem et al., 2016b). AMF can activate an antioxidant protection system, maintaining cell membrane stability by decreasing permeability and malondialdehyde (MDA) content in plants (Yang et al., 2014).

**Figure 1:**
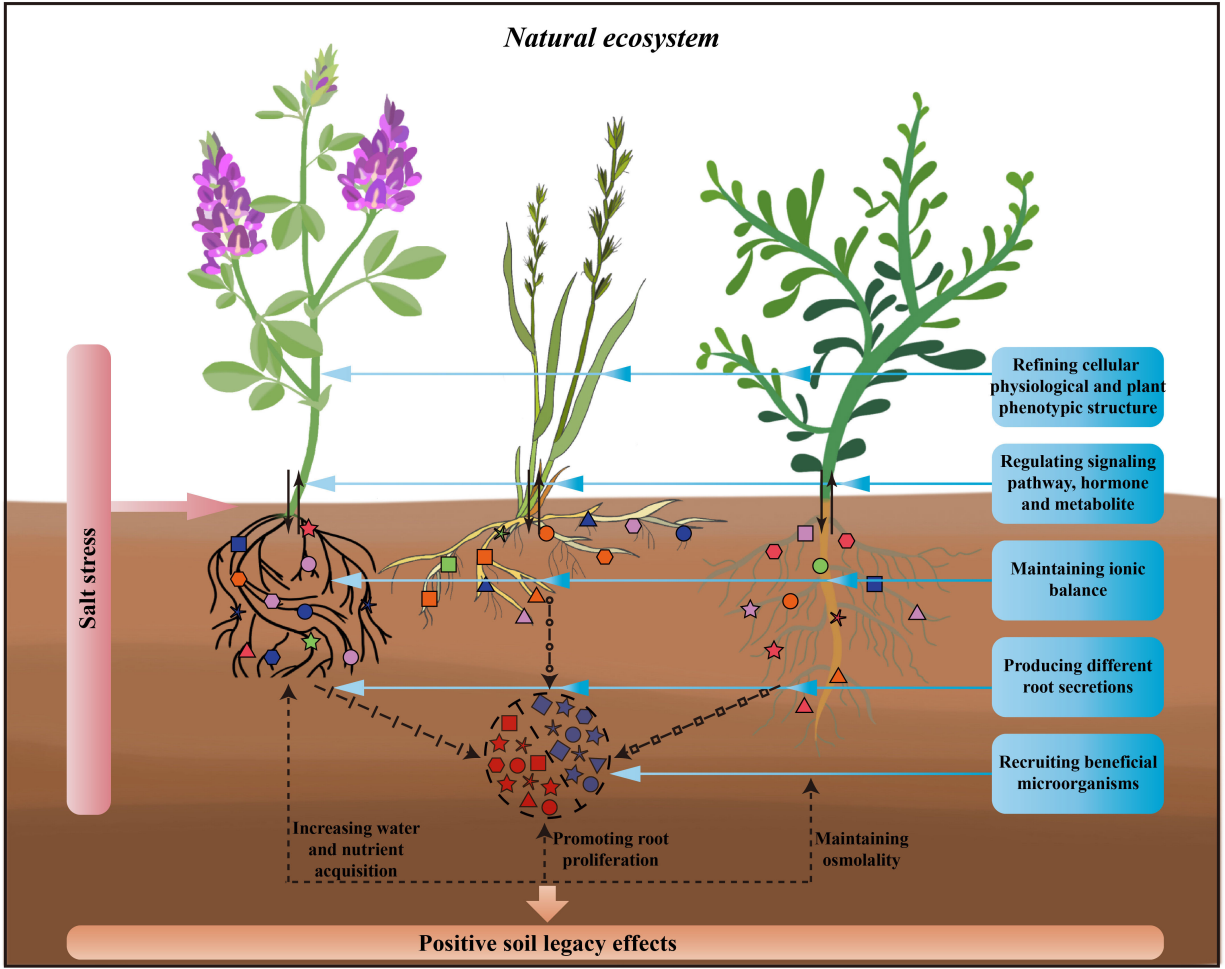
Processes of plant response to salt stress in natural ecosystems and possible soil legacy effects by plants. This figure shows, from left to right, three different plant functional groups, legume, grass, and forb, which respond simultaneously through above-ground and below-ground parts to salt stress. For above-ground parts of the plant, by refining cellular physiological and plant phenotypic structure, regulating signaling pathway, hormone and metabolite and thus responding to salt stress. For below-ground parts of the plant, by maintaining ionic balance, producing different root secretions, recruiting beneficial microorganisms and thus responding to salt stress. The response of above- and below-ground parts to salt stress simultaneously with increasing the plant’s own acquisition of soil water and nutrients, promoting plant root proliferation, and maintaining the osmolality of the plant as well as the rhizosphere, thus creating the positive soil legacy effects through this favourable response processes.

These complex processes converting salinity tolerance cannot be separated from the dynamic interactions between plants and microorganisms (Liu et al., 2022). In the context of climate change-induced stress, introducing new microbial taxa had been shown to improve plant survival in stressful environments, and plant tolerance can be predicted by the climatic history of the microbial community (Allsup et al., 2023). Building on this, plant-soil-microbe interactions in salt-stressed environments may result in a history of stress response for soil microbes and the soil environment, generating soil legacy effects that aid succeeding plants in overcoming salt stress (**Figure 1**; Li et al., 2021a; Jing et al., 2022).

## 4 Creating soil legacy effects to improve crop salt tolerance

Farmlands vulnerable to saline stress often experience extreme environmental conditions and undergo specific agricultural management practices. These practices include high surface evapotranspiration, low precipitation, elevated ambient temperatures, and the application of chemicals, along with heavy irrigation during production (Arora et al., 2018; Enebe and Babalola, 2018). In contrast to natural ecosystems, the production function of farmland directly determines its monoculture structure, resulting in low plant diversity and nutrient use efficiency, imbalanced dynamics between above-ground crops and below-ground soil food webs, and altered crop defense mechanisms (Savary et al., 2019). Crops cultivated in farmlands tend to prioritize growth over defense compared to their wild counterparts of the same species. This preference, combined with the monoculture structure, increases the likelihood of negative soil legacy effects between previous and succeeding crops (Mariotte et al., 2018). The multiple stresses of saline farmlands challenge the growth of crops and soil microbes, and there is a need to rethink how to create soil legacy environments that are conducive to crop growth, while optimizing agricultural practices and fostering sustainable methods to enhance soil health and crop (Li et al., 2014).

### 4.1 The use of plants to create soil legacy effects

**Figure 2:**
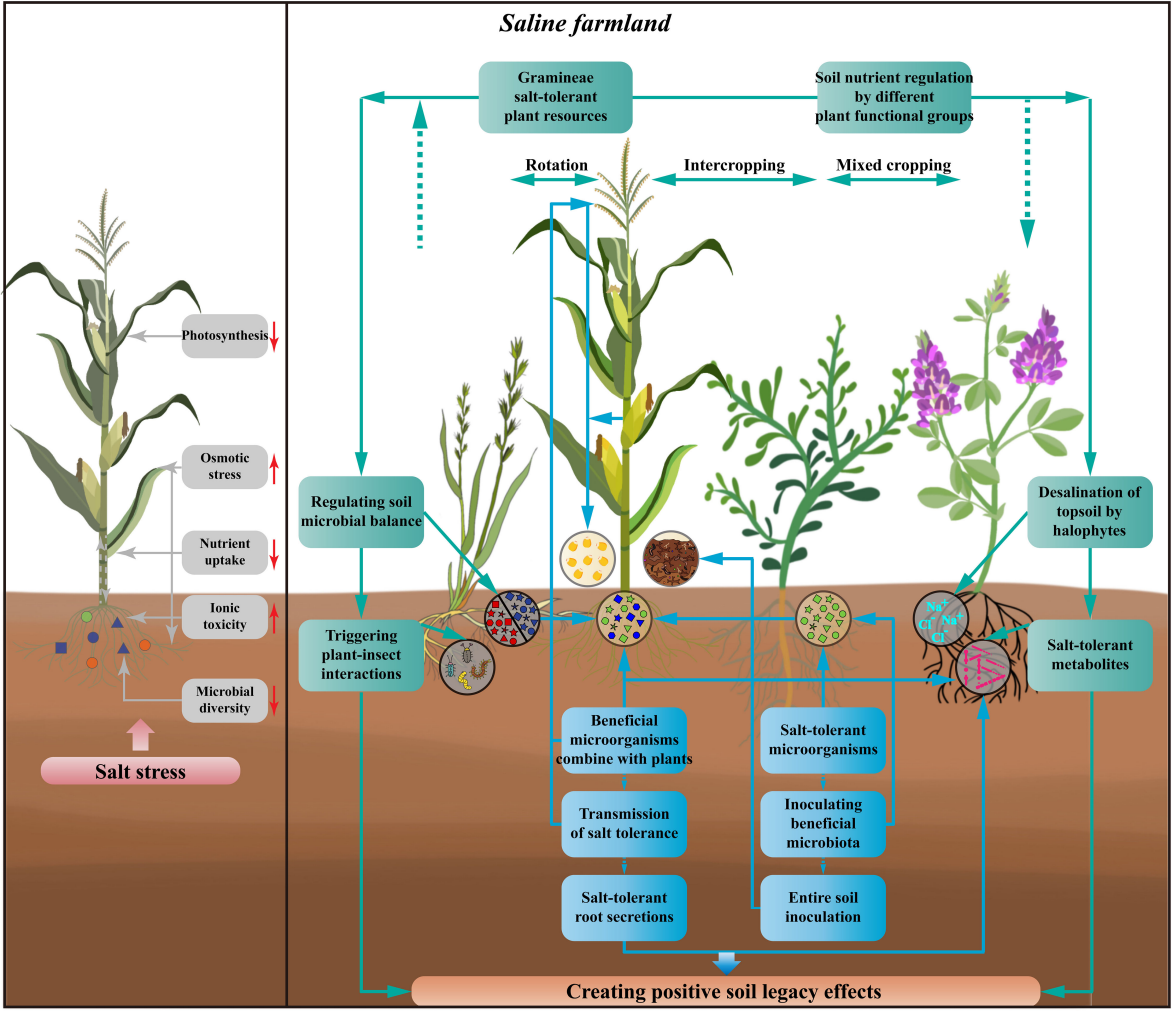
Effects of salt stress on crops and how to create soil legacy effects as well as improve crop salt tolerance in saline farmlands. The harmful effects of salt stress on crops include weakening crop photosynthesis, increasing osmotic stress, reducing crop nutrient uptake, adding ionic toxicity, and declining rhizosphere microbial diversity. By using halophytes, salt-tolerant plants, and plants of different functional groups, and developing the cropping patterns of rotating, intercropping, and mixed cropping with crops, the interactions between above- and below-ground parts of the plants can achieve the regulation of soil nutrients in saline farmlands, the desalination of surface soils, the secretion of salt-tolerant metabolite, and thus regulating the balance of soil microorganisms, as well as triggering the interactions between plants and insects. The improvement of salt tolerance in crops can also be achieved by screening for salt-tolerant microorganisms, inoculation with beneficial microbiota or entire soil inoculation. At the same time, new cultivation techniques could be used to combine the beneficial microorganisms directly with the plants and to transmit the tolerance. Crops with improved tolerance continue to produce salt-tolerant root secretions and to recruit beneficial microorganisms, thus creating an effective recycle of crop salt tolerance. All of these processes can create positive soil legacy effects through beneficial interactions between the above-ground and below-ground parts of the crop and influence succeeding crop.

The productivity constraints of saline farmlands primarily result from the highly stressful environment directly impacting the growth of aboveground crops. Most staple crops in agricultural production, such as maize, wheat, and rice, show high sensitivity to salinity stress. This sensitivity manifests itself in increased crop water loss, plant and fruit wilting, reduced crop photosynthesis, lowered carbon fixation, inhibited crop nutrient uptake, and slowed growth (Atta et al., 2023). To overcome the production bottlenecks in saline farmlands, it is necessary to harness biological resources with inherent salt tolerance found in natural environments. Additionally, establishing positive soil legacy effects through the introduction of specific plant species and plant functional groups is crucial (**Figure 2**).

#### 4.1.1 The use of salt-tolerant biological resources

One feasible approach is to utilize the ability of halophytes and salt-tolerant plants in the natural environment. Firstly, some halophytes can absorb salt ions, and they are effective in reducing surface soil salinity while fighting against the increase in ion levels in tissue cells through leaf succulence (Song and Wang, 2015). Halophytes also dissolve calcium in the soil through root respiration, where calcium ions replace sodium ions in the cation-exchange complex, and ultimately improve soil physical properties in the plant’s root zone (Qadir et al., 2005). Desalinated soils resulting from these processes contribute favorably to the subsequent growth of plants. Secondly, both halophytes and salt-tolerant plants boast robust root systems with strong penetration and water-holding capacity, thus enhancing soil structure (Silva et al., 2016). This improvement increases soil permeability and water retention post-planting, with the positive effects on soil structure persisting over an extended period (Liang and Shi, 2021). Finally, certain salt-tolerant plants, such as the forage crop sweet sorghum, can develop salt tolerance through hormonal signaling and secondary metabolites (Chen et al., 2022). Notably, stress-induced plant secondary metabolites have demonstrated legacy effects on succeeding plant growth by manipulating the composition of soil microbiome (Hu et al., 2018). Consequently, the utilization of halophytes and salt-tolerant plants presents opportunities to desalinate saline farmlands, improve soil conditions, or directly leverage the soil legacy effects created by the metabolites they produce to enhance crop resilience to salinity.

#### 4.1.2 Introducing plant functional groups into crop rotation systems

The soil legacy effects observed in natural ecosystems, facilitated by specific functional groups of plants, can significantly impact succeeding plants (Bezemer et al., 2006). This insight has inspired the development of effective cropping patterns for saline farmlands, especially considering that traditional monoculture patterns have contributed to soil resource depletion and decreased farmland productivity (Guo and Zhou, 2022). Grasses, have a solid research base in the field of ecology, known for carbon sequestration, nutrient cycling and improved soil stability (Franzluebbers, 2012; Hanamant et al., 2022). As the understanding of grassland ecosystem functioning continues to improve, forbs, representing a large proportion of species and functional richness, have also been recognized for their stress tolerance, indication of overgrazing, and maintenance of insect diversity (Siebert et al., 2021). Legumes, aside from being high-quality food and forage resources, are consistently recognized for sequestering nutrients and increasing diversity in cropping systems (Stagnari et al., 2017).

The crop rotation system of grasses and crops increased soil organic matter and earthworm numbers, resulting in improved soil structure compared to conventional crop rotations (Van Eekeren et al., 2008). This legacy effect of the grasses’ influence on soil properties, then, increased the yield and seed nitrogen content of succeeding crops (Christensen et al., 2009). Legumes are even more beneficial to agricultural production by providing diverse services. One aspect is that the nitrogen-fixing capacity of legumes can continually increase the nitrogen yield of succeeding crops (Fox et al., 2020). Moreover, the growth process of legumes releases organic acids and other compounds, directly activate nutrients and indirectly promote the activity of soil microorganisms, thus increasing crop yields and soil fertility (Latati et al., 2016). Studies have shown that the deposition of rhizosphere nitrogen in legumes accounts for 70% of the total plant nitrogen (Fustec et al., 2010). These deposited nitrogens have mechanisms for transfer to other crops, affecting agricultural production potential. Although there are fewer practices on the involvement of forbs in crop rotation, studies have shown that forbs are rather less affected by changes in nutrient conditions than grasses due to their ability to store nutrients in their roots (Herz et al., 2017). Forbs are also important for maintaining the diversity of arthropods in the environment and some forb communities are more resistant to herbivores (Potts et al., 2010; Van Coller et al., 2018). Therefore, introducing these plant functional groups, such as grasses, forbs, and legumes, during crop rotation can strategically change soil nutrient levels or indirectly regulate the biotic and abiotic environment of saline farmlands.

Moreover, grasses and forbs exhibit different abiotic stress tolerance mechanisms and growth strategies. Due to obvious differences in growth, development and physiological structure between grasses and forbs, applying knowledge of forbs to improve salt tolerance in major cereal crops becomes challenging (Tester and Bacic, 2005). Meanwhile, the ability of grasses to accumulate salt ions in shoots and leaves may be weaker than that of forbs due to fewer salt glands (Semenova et al., 2010). So, although the planting of forbs like *Suaeda salsa* can effectively reduce soil salinity, it is difficult to apply the mechanism of salt ion accumulation and succulence in shoots of forbs to crops of grasses.

However, Poaceae, particularly within the functional group of grasses, has a unique history of salt tolerance, including major halophytic taxa identified as sources of halophytes (Flowers et al., 1986). Compared to the forbs, grasses usually maintain ion levels in aboveground tissues by limiting sodium uptake, having high potassium/sodium selectivity, and efficient potassium utilization, essential for survival under saline conditions (Flowers and Colmer, 2008). Many wild-type grasses are naturally tolerate to salt stress (Landi et al., 2017). For example, the study found that its close wild relatives *Tripsacum dactyloides* and *Zea perennis* both showed strong salt tolerance compared to maize (Li et al., 2023b). The leaf surface of wild rice, *Porteresia coarctata*, can excrete salts, maintaining intercellular ion concentrations and lower sodium to potassium ratios (Sengupta and Majumder, 2010). Grasses have been reported to produce positive soil legacy effects by altering soil microbial communities, influencing nutrient transfer, and even triggering interactions between above-ground plants and insects (Kos et al., 2015; Cortois et al., 2016; Schmid et al., 2021). Also, the ionic changes that occur in grasses during salt tolerance are closely related to their rhizosphere microorganisms (Hamdia et al., 2004; Paul and Lade, 2014). Thus, by introducing plant functional groups into the crop rotation system and combining their different ecological functions and salt-tolerate characteristics, positive soil legacy effects can be generated. This provides broader thinking for the improving the soil environment in saline farmland and enhancing of crop salt tolerance.

#### 4.1.3 Introducing plant functional groups into crop intercropping system

The combination of plant functional groups within the same time and space can exert a significant influence on succeeding crops. One notable example is the legume and grass forage matching system, a typical forage mixing approach where the growth of grasses synergistically enhances both the symbiotic nitrogen fixation of legumes and the competitive nitrogen uptake of grasses (De Deyn et al., 2012; Suter et al., 2015). Beyond improving soil nutrient use efficiency, the extended growing period of mixed legumes and grasses also helps suppress topsoil salt accumulation, thereby enhancing soil quality (Li et al., 2021b).

While there are fewer studies on crop tillage systems and salt tolerance, similar to forage mixes, crop intercropping can weaken the negative impacts of saline farmland and may have legacy effects on succeeding crops. Firstly, intercropping systems increase the biodiversity of farmland ecosystems by direct introducing companion plants, such as differential crops or salt-tolerant plants, which provide services for saline farmland and the main crop (Yang et al., 2021). The introduction of different plants diversifies the rhizosphere environment, and the recruited microbial community can promote nutrient cycling, salt transformation, and degradation in the soil, thereby alleviating the damage of the saline environment to the crops. For example, the introduction of legumes can improve intercropping system resilience and resource use efficiency by enhancing crop growth and tolerance to abiotic stresses through root distribution, vegetative cover, and nutrient activation (Chamkhi et al., 2022). Furthermore, the intercropping of the halophyte *Suaeda salsa* with maize significantly transferred more sodium ions to the rhizosphere of *Suaeda salsa*, thereby reducing the salt content of the maize rhizosphere (Wang S. et al., 2021). Regarding the rhizosphere enrichment by intercropping systems, it was shown that legume-grass crop intercropping (maize/faba bean) increased the abundance of rhizobia and reduced pathogens in the soil. The soil legacy effects it produced could be one of the reasons for the observed yield advantage in intercropping systems (Wang et al., 2020). Particularly under salt stress, the beneficial microorganisms recruited by the intercropping system (sorghum/peanut) achieved increased crop tolerance by altering the composition and content of metabolites (Shi et al., 2023). Therefore, the potential positive soil legacy effects of salt-tolerant forage mixtures and salt-tolerant crops of different functional groups can help to develop efficient intercropping systems for saline farmlands.

### 4.2 The use of soil microorganisms to synergistically create soil legacy effects

The presence of soil microorganisms in natural ecosystems depends on the soil environment, chemical signals provided by plants and nutrient resources (Bai et al., 2022). In response to the direct release of stress-responsive signals and compounds in plants, the associated soil microorganisms undergo specific changes (Hartman and Tringe, 2019). These changes are closely related to plants, especially alterations in rhizosphere microorganisms, and are critical to support the growth and recovery potential of plants under stress (Park et al., 2023). Salt-tolerant microorganisms capable of thriving and multiplying in high-salt environments, directly aiding plants in tolerating salt stress through their salt-tolerance mechanisms (Sharma et al., 2015; Wang R. et al., 2021). Plants in traditional environments, when confronted with salt stress, also respond by directly recruiting beneficial microorganisms through root secretions (Kumar et al., 2023). Furthermore, the mechanism by which soil microorganisms regulate plant salt tolerance also involves osmotic regulators, nutrients and soluble salts they provide to plants. These pathways can indirectly influence plant hormones and metabolism, stimulate plant growth and help plants overcome salt stress (Glick, 2012; Shrivastava and Kumar, 2015). These actions not only alleviate the negative effects of salinity but also establish soil legacy effects that confer tolerance to succeeding plants (see **Figure 2**; Zhalnina et al., 2018; Otlewska et al., 2020). Considering this, the question arises: How can we apply the direct and indirect effects of soil microorganisms on plant salt tolerance to saline farmland? What measures can be taken to sustain these positive effects in the farmland?

#### 4.2.1 Direct utilization of soil microorganisms

Soil microorganisms play a crucial role in defending against saline stress, and saline soils serve as a significant source of salt-tolerant microorganisms (Zhang et al., 2023). Current research has successfully isolated several culturable salt-tolerant strains. For instance, 70% of the culturable strains of the root endophyte from the coastal perennial grass *Festuca rubra* exhibit salt tolerance (Pereira et al., 2019). The core microorganisms of the rhizosphere of *Suaeda salsa* have been found to harbor genes encoding salt stress adaptation and nutrient solubilization processes (Yuan Z. et al., 2016). Microbial inoculation is a direct method of utilizing these specialized salt-tolerant microbial resources, which can be applied to enhance plant salt stress adaptation and promote growth. Studies have demonstrated that inoculation with the salt-tolerant endophyte *Sphingomonas prati* significantly increases the salt tolerance of *Suaeda salsa* by improving the antioxidant enzyme system (Guo et al., 2021). *Curvularia* sp., isolated from *Suaeda salsa*, can establish a beneficial symbiotic relationship with poplar and promote its growth (Pan et al., 2018). Moreover, the inoculation of salt-tolerant microorganisms has been gradually extended to major crops, including soybean, maize, wheat, and peanut. Its positive effect in mitigating salt stress has been consistently verified in numerous indoor simulation experiments (Ramadoss et al., 2013; Goswami et al., 2014; Zerrouk et al., 2016; Khan et al., 2019; Shabaan et al., 2022).

In addition to the salt-tolerant microbial resources associated with saline soils and halophytes, salt stress is alleviated by the recruitment of beneficial microorganisms to the rhizosphere of plants when they face with salt stress in normal environments (Ilangumaran and Smith, 2017; Santoyo, 2021). For example, it has been shown that 1-aminocyclopropane-1-carboxylate (ACC), a stress-related amino acid in plants, can reshape the soil microbiome, enhancing plant tolerance to salinity stress (Liu et al., 2019). In addition, rice influences rhizosphere microorganisms by producing metabolites such as salicin and arbutin, enabling rhizosphere microorganisms associated salt stress tolerance (Lian et al., 2020). Moreover, beneficial rhizosphere microorganisms in plants can not only enhance salt-tolerant properties but also synergistically improve plant responses to salt stress by altering physiological growth processes, including seed germination, morphological structure, and biomass accumulation and partitioning (Pan et al., 2020). Regarding the inoculation of beneficial microbial strains to help crops tolerant salinity, studies have demonstrated that inoculation with *Pseudomonas flavescens* D5 strain effectively increased the biomass and antioxidant enzyme activities of barley, while reducing the adverse effects of salt stress on barley (Ignatova et al., 2022). Inoculation of candidate strains of *Azotobacter* has also been found to increase the potassium-sodium ratio, polyphenol and chlorophyll content, and decrease proline concentration in maize, thereby alleviating salt stress in maize by integrating multiple mechanisms (Rojas-Tapias et al., 2012).

Indeed, successful microbial inoculation often requires a combination of strains rather than a single strain to enhance the sustainability of its impact on (Verbruggen et al., 2012; Finkel et al., 2017). Notably, double inoculation with *Rhizobium* and *Pseudomonas* has been observed to elicit positive adaptive responses in alfalfa under salt stress (Younesi et al., 2013). Similarly, dual inoculation of plant growth-promoting bacteria with *Bradyrhizobium* strains has proven more effective in enhancing salt tolerance in soybean, reducing salt-induced ethylene production, and improving nutrient uptake (Win et al., 2023). Further studies have found that inoculation with species-specific microbiomes or whole-soil inoculation can assist plants in coping with various biotic and abiotic stresses (De Vries et al., 2020; Ma et al., 2020; Trivedi et al., 2020). The introduction of microbiomes or the whole-soil achieves more complex ecological functions by coordinating microbial interactions (Pineda et al., 2019; Trivedi et al., 2021), and it avoids the potential issue of single strains struggling to survive inoculation into foreign soil (Mallon et al., 2018). However, it is crucial to acknowledge the possibility that introducing exotic microbial communities may reshape functions within the native microbial community (Amor et al., 2020). Recent evidence suggests that the beneficial effects of microbial inoculation on plant growth are best explained as changes in native microorganisms rather than direct effects on plants (Hu et al., 2021). This underscores the importance of understanding the intricate interactions occurring within the microbial community and their influence on plant health and resilience.

While practical examples of microorganism inoculation for saline farmland improvement are limited, the concept of soil legacy effects suggests that enhancing saline farmland and crops can be achieved through microbial-mediated processes. By inoculating salt-tolerant microbial strains and communities of beneficial microorganisms, and even inoculating the entire soil including most microorganisms, it becomes possible to modulate crop responses to salt stress and enhance salt tolerance. Concurrently, synergistic changes with the inoculated microorganisms involve stress response-related metabolites and alterations in the crop rhizosphere environments. These changes encompass crop rhizosphere secretions, microbial metabolites, and native microbial communities. Their persistent influence on succeeding crop growth in the form of soil legacy effects contributes to ongoing salt stress mitigation in saline farmland. Thus, the application of microbial interventions holds promise for sustainable improvements in saline farmland and crop resilience (Cuddington, 2011; Trivedi et al., 2020).

#### 4.2.2 Indirect utilization of soil microorganisms

Alongside traditional plant- and microorganism-based methods for restoring saline farmlands, advanced modern agricultural techniques with their high efficiency and precision have also found application agricultural production (Varshney et al., 2011; Ahanger et al., 2017). Research has focused on integrating and applying the active components of rhizosphere exudates to soil microbial systems, revealing improvements in soil physicochemical environments and microbial communities associated with rhizosphere exudates. These improvements are speculated to have an impact on plant growth (Shi et al., 2011). Similar findings were observed in maize system, where a significant increase in bacterial density and altered metabolic potential in the maize rhizosphere after application of maize rhizosphere exudates (Baudoin et al., 2003). In terms of enhancing crop tolerance, research has shown that introducing the ability of releasing volatile organic compounds (VOCs) into maize varieties that do not release specific VOCs can reduce the threat of pests (Degenhardt et al., 2009). This suggests that the introduction of tolerant metabolites is not limited to rhizosphere exudates, and the application of below-ground volatiles, as well as other tolerant signals, offers additional possibilities for improving salt tolerance in crops on saline farmlands. The advances in agricultural technology have also inspired the exploration of beneficial root traits in wild relatives of crops, the introduction of which may solve the problems faced by saline farmlands (Preece and Peñuelas, 2020).

In the past decade, cultivation techniques have gradually emerged, pointing to the unique microbiome existing in plant seeds and how it spreads from generation to generation, aiding plants in adapting to their environment and increasing tolerance (Gopal and Gupta, 2016; Abdelfattah et al., 2023). In this context, delivering endophytes to the next generation of crops and ensuring the persistence of their tolerance has been achieved by combining relevant beneficial microorganisms with plants (Wei and Jousset, 2017). For example, a suspension of *Paraburkholderia phytofirmans* PsJN was sprayed in plots at the flowering stage of wheat in field experiment, and thus the maturation of its progeny plants was accelerated by the introduction of this endophytic bacteria into the flowers of the wheat parents (Mitter et al., 2017). The advantage of this approach lies in the ability of seed endophytes to avoid competition with native soil microorganisms, establishing closer interactions with the plant early on. While there is currently limited research related to this approach concerning salt tolerance in progeny plants, seed endophytes have long been shown to provide plants with tolerance against a wide range of stresses, participate in plant adaptation mechanisms, and enhance plant competitiveness (Samreen et al., 2021). Therefore, the use of these new bioculture techniques and the genetic mechanisms of plant microbes offer innovative avenues for improving saline farmland. These approaches are closely related to plant-microbe interactions and are centered around the concept of creating positive soil legacy effects.

Inspired by the mentioned approaches, microorganisms can be used indirectly, such as through the recruitment of microorganisms by plant rhizosphere exudates and intergenerational dissemination of beneficial microorganisms, to create positive soil legacy effects in saline farmland. However, it is acknowledged that microbial-related methods of creating soil legacy effects are imperfect, and their processes may introduce soil pathogens or other responsive substances, necessitating further in-depth research to explore safer methods of creating soil legacy effects (Jing et al., 2022).

## 5 Conclusion and future prospects

This paper provides a summary of the ways in which plants, in collaboration with soil microorganisms in natural ecosystems, jointly respond to salt stress. It suggests enhancing the salt tolerance of crops in saline farmlands through the perspective of soil legacy effects. The focus is on meeting the salt tolerance needs of crops by creating well-considered soil legacy effects. The paper explores both the direct use of plants and the synergistic use of soil microorganisms to establish positive soil legacy effects, offering innovative insights to boost production potential and improve the ecological environment of saline farmland. The emphasis lies on creating positive soil legacy effects through the selection of suitable salt-tolerant crops, the development of planting patterns with a rational match of crop functional groups, the inoculation of functional microorganisms, the inoculation of safe and efficacious soils, and the application of advanced agricultural technologies and bio-cultivation methods. This approach underscores the practical utility of crop-soil microorganism interactions in agricultural production. In addition to plants and associated soil microorganisms, the role of soil animals in constructing soil food webs is acknowledged. These soil animals, through direct or indirect interactions with microorganisms and plants, contribute to the cycling of soil nutrient resources, influencing soil ecosystem function (Du et al., 2018). Multi-trophic interactions between mycorrhizal fungi, fungus-eating protozoa, and nematodes in the soil can enhance crop nutrient uptake, crop yield, and tolerance (Jiang et al., 2020). This suggests that future studies can more precisely and directly leverage soil legacy effects to trigger positive tolerant responses by regulating specific species or soil fauna in the soil food web of saline farmlands, or even by controlling certain trophic levels.

## Author contributions

YM: Writing – original draft, Writing – review & editing. CZ: Funding acquisition, Writing – review & editing. YB: Funding acquisition, Writing – review & editing. CS: Funding acquisition, Writing – review & editing. FZ: Funding acquisition, Supervision, Writing – review & editing.
